# FRET Based Quantification and Screening Technology Platform for the Interactions of Leukocyte Function-Associated Antigen-1 (LFA-1) with InterCellular Adhesion Molecule-1 (ICAM-1)

**DOI:** 10.1371/journal.pone.0102572

**Published:** 2014-07-17

**Authors:** Sandeep Chakraborty, David Núñez, Shih-Yang Hu, María Pilar Domingo, Julian Pardo, Artashes Karmenyan, Eva M Gálvez, Arthur Chiou

**Affiliations:** 1 Institute of Biophotonics, National Yang-Ming University, Taipei, Taiwan; 2 Instituto de Carboquímica, CSIC, Zaragoza, Spain; 3 Immune Effector Cells Group, Aragón Health Research Institute, Biomedical Research Centre of Aragón, Zaragoza, Spain; 4 Department of Biochemistry and Molecular and Cell Biology, Facultad de Ciencias, University of Zaragoza, Zaragoza, Spain; 5 Aragón I+D Foundation, Government of Aragon, Zaragoza, Spain; 6 Nanoscience Institute of Aragón, Aragón I+D Foundation, University of Zaragoza, Zaragoza, Spain; 7 Biophotonics & Molecular Imaging Research Center, National Yang-Ming University, Taipei, Taiwan; CNR, Italy

## Abstract

The interaction between leukocyte function-associated antigen-1(LFA-1) and intercellular adhesion molecule-1 (ICAM-1) plays a pivotal role in cellular adhesion including the extravasation and inflammatory response of leukocytes, and also in the formation of immunological synapse. However, irregular expressions of LFA-1 or ICAM-1 or both may lead to autoimmune diseases, metastasis cancer, etc. Thus, the LFA-1/ICAM-1 interaction may serve as a potential therapeutic target for the treatment of these diseases. Here, we developed one simple ‘in solution’ steady state fluorescence resonance energy transfer (FRET) technique to obtain the dissociation constant (K_d_) of the interaction between LFA-1 and ICAM-1. Moreover, we developed the assay into a screening platform to identify peptides and small molecules that inhibit the LFA-1/ICAM-1 interaction. For the FRET pair, we used Alexa Fluor 488-LFA-1 conjugate as donor and Alexa Fluor 555-human recombinant ICAM-1 (D1-D2-Fc) as acceptor. From our quantitative FRET analysis, the K_d_ between LFA-1 and D1-D2-Fc was determined to be 17.93±1.34 nM. Both the K_d_ determination and screening assay were performed in a 96-well plate platform, providing the opportunity to develop it into a high-throughput assay. This is the first reported work which applies FRET based technique to determine K_d_ as well as classifying inhibitors of the LFA-1/ICAM-1 interaction.

## Introduction

The Leukocyte Function-associated Antigen-1 (LFA-1, also known as CD11a/CD18, or α_L_β_2_), a member of the integrin superfamily of cell surface adhesion molecules, is a heterodimeric type I transmembrane glycoprotein consisting of one α_L_-subunit (CD11a, 180 kD) and one β_2_-subunit (CD18, 95 kD) which are non-covalently associated with each-other [1]–[3]. These two subunits form an extracellular domain, which is further subdivided into 13-subdomains, and two short cytoplasmic tails [4]. LFA-1 is expressed exclusively on all leukocytes. The adhesion of leukocytes with other cells is mediated through the interactions of LFA-1 with its ligands, ICAM-1 (CD54) [5], [6], ICAM-2 (CD102) [7], ICAM-3 (CD50) [8], [9], ICAM-4 [10] or ICAM-5 (telencephalin) [11]. Among these, intercellular adhesion molecule-1 (ICAM-1), a transmembrane glycoprotein of Ig supergene family, shows the highest affinity towards LFA-1 [12]. It is composed of five extracellular Ig-like domains (domains 1–5, D1–5), a transmembrane domain, and a short cytoplasmic domain [13], [14]. Almost all kinds of nucleated cells including endothelial cells, epithelial cells and leukocytes express ICAM-1 [13]. Apart from LFA-1, ICAM-1 also serves as a receptor for another integrin Mac-1 [15], the human rhinovirus [16], coxsackie A21 virus [17], and malaria parasite *Plasmodium falciparum* [18].

ICAM-1 interacts with its counter receptor LFA-1 through the binding of its first Ig-domain (D1) with the I- (inserted) domain on the top of the N-terminus of α_L_ subunit of LFA-1 [19]. More specifically, the binding site of ICAM-1 is localized to a metal ion-dependent adhesion site (MIDAS) motif of the α_L_ subunit I-domain of the integrin [20]. Binding of divalent cations, such as Mg^2+^or Mn^2+^, to the MIDAS can activate LFA-1 and results in a high affinity interaction with its receptor. These divalent cations coordinate the five amino acids, Ser-139, Ser-141, Asp-237, Thr-206, and Asp-239, of the MIDAS and glutamate 34 in D1 of ICAM-1 to form a cation coordination complex to facilitate the LFA-1 and ICAM-1 interaction [21], [22].

This molecular interaction of the activated LFA-1 with ICAM-1 plays an important role in many physiological processes such as in the leukocyte-endothelial cell adhesion cascade, resulting in the extravasation of leukocytes to the site of inflammation and the formation of immunological synapse between leukocytes and an antigen presenting cell (APC) [23], [24]. However, the irregular expressions of LFA-1 or ICAM-1 or both have also been related to the specific pathologies of several autoimmune diseases such as multiple sclerosis (MS), leukocyte adhesion deficiency (LAD), thyroiditis, and insulin-dependent diabetes mellitus (IDDM) [25], [26]. For example, in MS, leukocytes show higher adhesion capacity than normal; in addition, over expression of LFA-1 is also observed, indicating that the LFA-1/ICAM-1 interaction may play a role in the adhesion of leukocytes to brain microvascular endothelial cells (MVEC) and ultimately in the disease pathology [27]. Cell adhesion molecules also mediate in viral budding and transfer. It has been demonstrated that high affinity form of LFA-1 facilitates viral entry and subsequently in the HIV syncytium formation [28]. Moreover, the LFA-1/ICAM-1 interaction induces the necessary cytokine release for the termination of tumor growth while lymphocytes interact with tumor cells; however, tumor cells with higher level of cell adhesion molecules are observed to form metastatic lesions [29], [30].

Based on these roles in several disease pathologies, the LFA-1/ICAM-1 interaction can serve as a potential therapeutic target in the development of new therapies for the autoimmune diseases, metastasis cancer, and viral diseases. For example, anti-ICAM-1 and anti-LFA-1 were used in combination to inhibit the LFA-1/ICAM-1 interaction to overcome the cardiac allograft rejection [31]. Furthermore, several antibiotic therapies were developed for the treatment of autoimmune diseases [32]. However, antibiotic therapies have serious side effects in humans due to their large molecular structure and nonhuman origin [33]. Thus, to overcome these difficulties associated with antibiotic therapies, small molecules, such as lovastatin, which specifically inhibits the interaction of ICAM-1 with LFA-1 [34], were also developed. The most important step towards developing inhibitory molecules to the LFA-1/ICAM-1 interaction, however, was the use of short peptides [35]. These peptides are capable of binding to the specific sites of the target proteins and eventually can interfere with their activity [36], [37]. Hence, a better understanding of the structure, function, and the mechanism of the interaction of ICAM-1 and LFA-1 may further lead to novel therapeutic drug discoveries and therapeutic tools.

Quantitative analysis of protein-protein interactions *in* *vitro* is of fundamental importance in understanding these complex biochemical processes. Among several methods that are available in this context, Förster/Fluorescence resonance energy transfer (FRET) technique has been widely used *in* *vitro* and *in* *vivo* to study protein-protein interactions [38], [39]. FRET is a highly distant-dependent process where a fluorescent molecule (donor), in its excited state, transfers energy non-radiatively to another molecule (acceptor) through dipole-dipole interactions [40]. Since FRET efficiency is proportional to 1/r^6^, where “r” is the distance between the donor and acceptor, the FRET signal provides a high degree of spatial sensitivity (between 1 and 10 nm) and signal specificity; hence, it has been developed and well recognized as a very powerful tool for the study of protein-protein interactions [40]. Moreover, FRET is highly suitable for both spectroscopic and imaging for static and real-time analysis. FRET has many advantages over other current techniques used for quantitative protein interaction studies, such as surface plasmon resonance (SPR) [41], isothermal titration calorimetry (ITC) [42], or radio-labled ligand binding assay [43]. In addition, FRET-based studies also complement other single molecule fluorescence–based techniques such as fluorescence correlation spectroscopy (FCS) [44], [45].

FRET measurements can be done in aqueous or solution phase, similar to the environment in physiological conditions. Besides, FRET measurements require only general fluorescence spectrometers or microscopes compared to other mentioned methods which need sophisticated instruments. Moreover, FRET measurements do not require any special conjugation or orientation of the proteins over other surfaces, other than conjugating the proteins with fluorophores, which in general does not affect the protein functional properties. Due to these advantages, quantitative FRET assays have been developed to study protein-protein interactions [46], [47]. However, an earlier obstacle in successful development of these assays was to extract the FRET emission signal at the acceptor emission wavelength from a mixed emission spectrum which contains the unquenched donor emission, direct acceptor emission and the actual FRET emission signal. Recently, Song et al. [48], [49] developed a theoretical and experimental procedure to overcome this obstacle and obtained the dissociation constant (K_d_) of the interaction between SUMO 1 and Ubc9. In this method, correlation of donor and acceptor emissions were used to obtain the absolute fluorescence signal contributions (due to unquenched donor, direct acceptor and FRET emission signal) at the acceptor emission wavelength in one single assay [49].

In this study, we have developed a steady-state ‘in-solution’ based quantitative FRET assay to determine the dissociation constant (K_d_) of the interaction between LFA-1 and ICAM-1. The FRET emission signal was obtained from a single step process. The interaction was studied using a chimeric molecule consisting of the amino-terminal first two Ig domains of human ICAM-1 (D1-D2) fused to IgG1 Fc region (D1-D2-Fc) [50]. In our FRET assay, Alexa Fluor 488-LFA-1 served as the donor, and Alexa Fluor 555-D1-D2-Fc, the acceptor. Our results show that the human recombinant D1-D2-Fc interacts with LFA-1 with a K_d_ of 17.93±1.34 nM. Moreover, we used this LFA-1 and D1-D2-Fc interaction to develop an ‘in-solution’ FRET based screening assay to measure the inhibition (to the LFA-1/ICAM-1 interaction) of LFA-1 derived short peptides (CD11a_237–261_, CD11a_441–465_, and CD11a_456–465_) in terms of their inhibition efficiency, derived from the corresponding FRET efficiency. In addition, we have determined the optimal fluorophore to protein (F/P) ratios and acceptor to donor (A/D) fluorophore ratios to develop the screening platform to specifically detect the inhibitors of the LFA-1/ICAM-1 interaction. The developed steady-state FRET based assay can further be used to study the LFA-1/ICAM-1 interaction in several physiological conditions *in* *vitro* and also have the potential to be developed into a high through-put screening (HTS) assay to identify potential inhibitors to LFA-1/ICAM-1 interactions as well as for other protein-protein interactions.

## Results and Discussion

**Figure 1:**
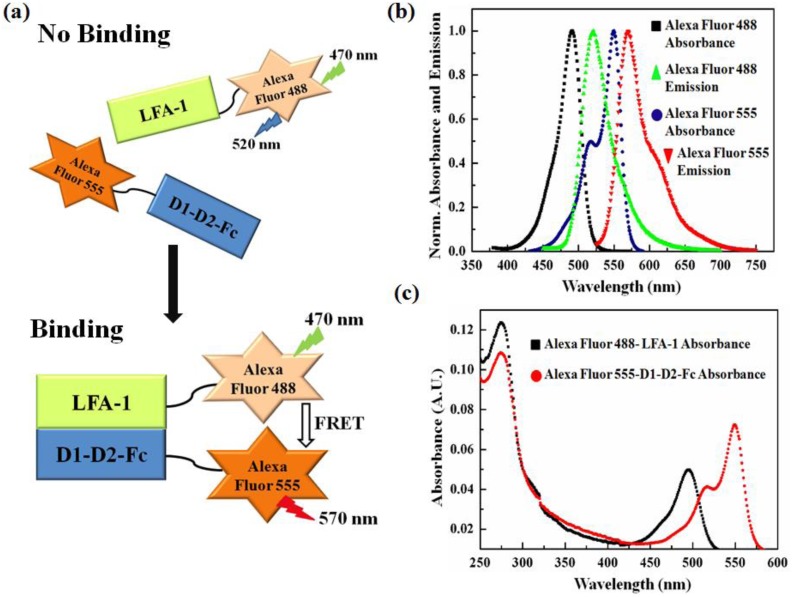
Illustration of the FRET assay. (a) A schematic diagram illustrating the phenomena of FRET with Alexa Fluor 488 conjugated LFA-1 as the donor and Alexa Fluor 555 conjugated D1-D2-Fc as the acceptor. When there is no binding between LFA-1 and D1-D2-Fc, excitation of donor at 470 nm leads to 520 emission peak only. However, when there is binding between LFA-1 and D1-D2-Fc, excitation at 470 nm leads to acceptor emission at 570 nm, due to FRET. (b) Normalized absorbance and emission spectra of Alexa Fluor 488 and Alexa Fluor 555. (c) UV- visible absorption spectra for fluorophore-protein conjugates (for the determination of the molecular concentration of fluorophores and proteins).

In our FRET assay, the conjugated fluorophore-protein pair of Alexa Fluor 488-LFA-1 served as the donor, while Alexa Fluor 555-D1-D2-Fc as the acceptor. Both fluorophores are amine-reactive reagents which can react with non-protonated aliphatic amine groups, including the amine terminus of proteins and the ε-amino groups of lysines. The scheme for FRET measurement is illustrated in Fig. 1(a). The normalized absorption and emission spectra for Alexa Fluor 488 and Alexa Fluor 555 were obtained to confirm the fluorescence properties of the fluorophores in our experimental conditions [Fig. 1(b)].

### Determining the fluorophore to protein (F/P) molar ratio

The fluorophore to protein (F/P) molar ratio was obtained for each of the Alexa Fluor 488-LFA-1 and Alexa Fluor 555-D1-D2-Fc conjugates. Based on the UV-visible absorption spectra for the fluorophore-protein conjugates [Fig. 1(c)], the F/P molar ratios were calculated to be 1.48±0.09 and 3.14±0.27 for Alexa Fluor 488-LFA-1 and Alexa Fluor 555-D1-D2-Fc, respectively.

The knowledge of F/P molar ratios was utilized to calculate the actual concentrations of donors and acceptors in the solutions. Moreover, the FRET efficiency is highly dependent on the F/P ratio. In general, higher F/P ratio should enhance resonance energy transfer due to the increase in the overlap integral; i.e. the increase in the number of acceptors per donor increases the effective acceptor extinction coefficient, which in turn proportionally improves the effective overlap integral for single donor-multiple acceptor complexes and vice-versa [40], [51]. However, high F/P ratio may also lead to concentration dependent quenching, and consequently, low sensitivity. Moreover, in our case the fluorophores were conjugated randomly to the proteins through their binding with the primary amines. Hence, higher F/P ratio may contribute to a significant background noise. On the other hand, low F/P ratios will lead to very low signals which may not be easy to differentiate from the background noise. Recent theoretical and experimental studies have also indicated that FRET efficiency can be significantly increased by optimizing the F/P ratio, especially when the energy transfer takes place to multiple acceptors [52], [53]. Following these facts, we optimized the conjugation protocol to obtain F/P ratios for our fluorophore-protein conjugates to have strong FRET activity, and also for the subsequent determination of the dissociation constant (K_d_) of the LFA-1/D1-D2-Fc interaction as well as in screening assay development to classify inhibitors of the interaction.

### Ascertaining the FRET activity

**Figure 2:**
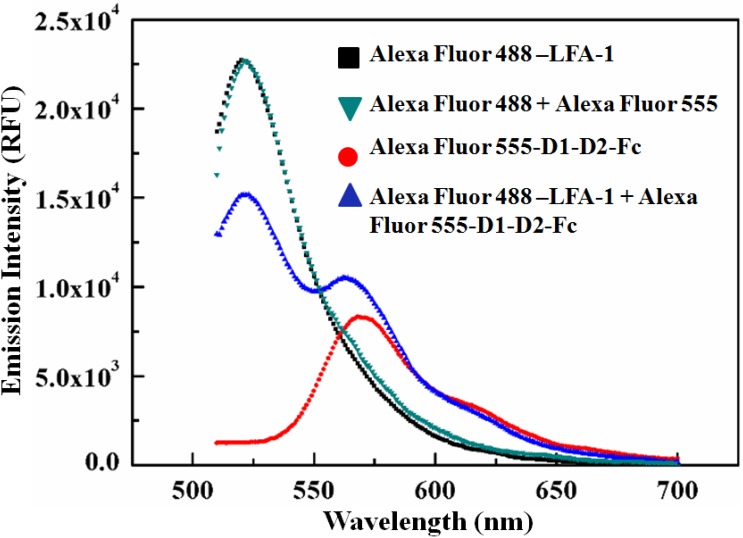
Ascertaining the FRET activity between Alexa Fluor 488-LFA-1 and Alexa Fluor 555-D1-D2-Fc. Fluorescence emission spectra of individual fluorophore-protein conjugates, Alexa Fluor 488-LFA-1 (100 nM) and Alexa Fluor 555-D1-D2-Fc (100 nM), and a mixture of Alexa Fluor 488-LFA-1 (100 nM) and Alexa Fluor 555-D1-D2-Fc (100 nM) are compared. A sensitized acceptor emission at 570 nm and a donor quenching at 520 nm are observed in the emission spectra of the FRET mixture, confirming FRET. For control, the emission spectrum of the mixture of the dyes (Alexa Fluor 488+ Alexa Fluor 555), 100 nM each is also shown. That fact that no emission peak appeared at 570 nm for Alexa Fluor 555 when only the dye mixture (Alexa Fluor 488+ Alexa Fluor 488) was excited at 470 nm, whereas a prominent acceptor sensitized peak was observed for the FRET mixture (Alexa Fluor 488-LFA-1+ Alexa Fluor 555-D1-D2-Fc) indicates that the random FRET between the free dye molecules can be neglected in our study. All the spectra were obtained under the excitation of 470 nm. The gain of the spectrofluorometer was set at 100 (manual). The excitation and the emission bandwidths were fixed at 9 nm (for 316–850 nm excitation range) and 20 nm (for 280–850 nm emission range) for all the measurements. The fluorescence emissions were recorded with an integration time of 20 µs (more details on Table S1).

In developing our spectral FRET assay, LFA-1 and D1-D2-Fc were covalently labelled with small organic molecule fluorophores, Alexa Fluor 488 (λ_ex_ = 488 nm; λ_em_ = 520 nm) and Alexa Fluor 555 (λ_ex_ = 555 nm; λ_em_ = 570 nm), respectively. To ascertain the FRET activity between the fluorophore-protein conjugates, the emission spectra of Alexa Fluor 488-LFA-1 (100 nM), Alexa Fluor 555-D1-D2-Fc (100 nM), the equimolar mixture of Alexa Fluor 488-LFA-1 and Alexa Fluor 555-D1-D2-Fc as well as the equimolar mixtures of Alexa Fluor 488 and Alexa Fluor 555 were obtained under 470 nm excitation and compared. Here, and in the rest of this article, the concentration values represent the concentration of the protein in the fluorophore-protein conjugate solution, and not the fluorophore concentration. The key parameters of the fluorescence multiplate reader set for these measurements (to obtain the experimental results shown in Fig. 2) are listed in Table S1. From Fig. 2 it is clearly observed that the donor emission intensity is quenched around 520 nm; whereas an increase in the acceptor emission is observed around 570 nm for the fluorophore-protein conjugate mixture. This sensitized emission intensity of the acceptor is significantly higher than that corresponding to the direct emission of Alexa Fluor 555-D1-D2-Fc. These prominent spectral changes indicate the non-radiative energy transfer between Alexa Fluor 488 and Alexa Fluor 555 in the fluorophore-protein conjugate mixture and the binding between LFA-1 and D1-D2-Fc. We noticed that the count rate was very high for the donor alone (at 520 nm), in comparison with the corresponding value for the acceptor alone (at 570 nm); this can be attributed to the fact that the samples (i.e., the donor alone, the acceptor alone, as well as the FRET mixture) were excited at 470 nm, which is the excitation maxima of donor. Moreover, the large difference in the quantum yields of Alexa Fluor 488 (0.92) and of Alexa Fluor 555 (0.10) [54] may also contribute to this result.

### Steady-state FRET binding assay to determine the dissociation constant (K_d_) of the interaction between LFA-1 and D1-D2-Fc

**Figure 3:**
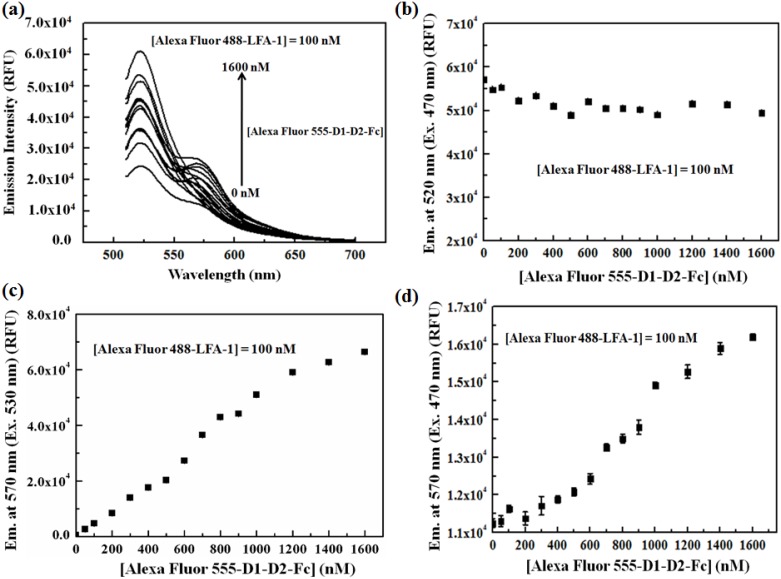
Determination of the FRET emission signal at 570 nm. (a) Fluorescence emission spectra (when excited at 470 nm) of Alexa Fluor 488-LFA-1 and Alexa Fluor 555-D1-D2-Fc mixtures, wherein the Alexa Fluor 488-LFA-1 concentration was fixed at 100 nM and that of Alexa Fluor 555- D1-D2-Fc was varied from 0 to 1.6 µM; (b) Emission signal of the donor (F_D_) in the mixture at 520 nm when excited at 470 nm; (c) Emission signal of Alexa Fluor 555 at 570 nm (F_A_) in the mixture when excited at 530 nm; (d) Total emission signal of the mixture at 570 nm when excited at 470 nm. From (b), we see that the quenching of the donor is negligible when the acceptor concentration exceeds ∼500 nM, which indicates that the increased concentrations of acceptor gradually saturate the number of donor binding pairs.

In determining the equilibrium dissociation constant (K_d_) between the interacting protein pairs, LFA-1 and D1-D2-Fc, from the FRET binding assay, several concentrations of the acceptor conjugate, Alexa Fluor 555-D1-D2-Fc, were added to a fixed concentration of the donor conjugate, Alexa Fluor 488-LFA-1. The donor conjugate concentration was kept constant at 100 nM while the acceptor conjugate concentration was varied from 0 upto 1.6 µM. These FRET mixtures, with different donor-to-acceptor ratios, were excited by 470 and 530 nm. The fluorescence emission spectra for the FRET assay, excited at 470 nm, are shown in Fig. 3(a). Each spectrum shows two distinct peaks, one at 520 (due to unquenched Alexa Fluor 488, F_D_), and the other at 570 nm (F_DA_). The peak intensity at 570 nm has contributions from unquenched donor emissions, direct acceptor emission and the emission of acceptor due to FRET (F_FRET_). However, when the FRET mixture was excited with 530 nm, only a single peak corresponding to the direct emission of the acceptor (F_A_) was observed.

Thus, to obtain the absolute maximum FRET emission signal at 570 nm (F_FRETmax_), we need to quantify each contribution from the donor and acceptor emissions as well as the FRET emission signal (F_FRET_). Fig. 3 [b] shows the donor emissions (F_D_) when the FRET mixture was excited with 470 nm. It is clearly observed that the direct emission of Alexa Fluor 488-LFA-1 decreased as the concentrations of the Alexa Fluor 555-D1-D2-Fc was increased from 0 upto 1.6 µM, as more donors were bound to the acceptors when the concentration of the later was increased. However, the emission at the 570 nm from the Alexa Fluor 555-D1-D2-Fc (F_A_) increased steadily as the FRET mixture was excited with 530 nm [Fig. 3(c)]. The total emission signal at 570 nm (F_DA_), when the FRET mixture was excited at 470 nm, also increased with the increase in the acceptor concentration [Fig. 3(d)].

**Figure 4:**
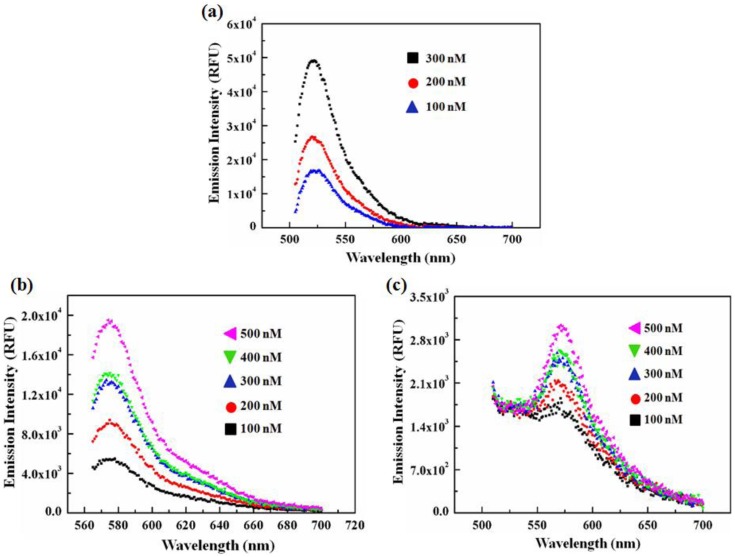
Determination of the ratio constants, *a* and *b*. (a) To obtain ***a,*** fluorescence emission spectra of Alexa Fluor 488-LFA-1 alone at three different concentrations (100, 200, and 300 nM), excited with 470 nm, were obtained. To calculate ***b***, fluorescence emission spectra of Alexa Fluor 555-D1-D2-Fc only at several concentrations (100, 200, 300, 400, and 500 nM) upon excitation at 530 nm (b) and 470 nm (c) were obtained.

In general, the direct emission of Alexa Fluor 488-LFA-1 at 570 nm is expected to be proportional to its emission at 520 nm when excited at 470 nm. This proportionality factor was denoted as “*a*” and defined as the ratio of the emission intensity at Alexa Fluor 488-LFA-1 alone at 570 nm to that at 520 nm when excited at 470 nm. To determine the ratio factor, “*a*”, several concentrations of Alexa Fluor 488-LFA-1 were prepared and their corresponding emission spectra were obtained [Fig. 4(a)]. From these spectra and following the definition, the value of “*a*” was found to be 0.129±0.015. Likewise, it is also logical to expect that the direct emission of Alexa Fluor 555-D1-D2-Fc at 570 nm when excited at 470 nm is proportional to its emission at 570 nm when excited at 530 nm and this ratio factor was denoted as “*b*”. To obtain “*b*”, a series of concentrations of Alexa Fluor 555-D1-D2-Fc alone was prepared and their emission spectra were obtained upon excitation at 530 [Fig. 4(b)] and 470 nm [Fig. 4(c)]. The calculated value of “*b*” was 0.101±0.008. Now, multiplying F_D_ with “*a*” and F_A_ with “*b*” will give the actual direct emission contribution of the donors and acceptors at 570 nm when the FRET mixture is excited with 470 nm, which was used in the subsequent determination of the dissociation constant.

#### Determination of the dissociation constant (Kd)

**Figure 5:**
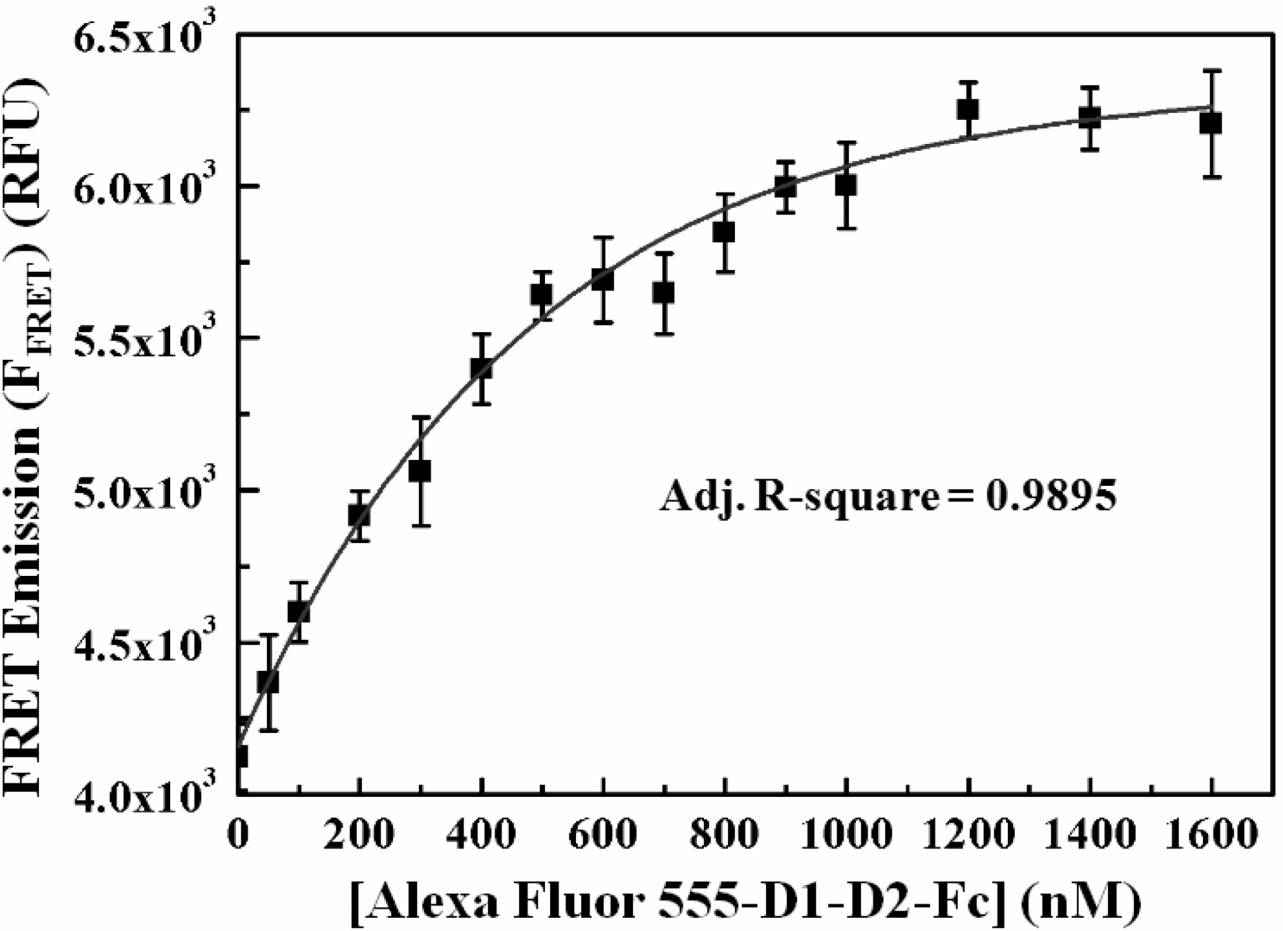
Determination of dissociation constant (K_d_) and maximum FRET emissions (F_FRETmax_) signal. The plot shows the fitting curve of FRET emission signal with equation (6). The F_FRETmax_ and the corresponding K_d_ of the LFA-1/D1-D2-Fc interaction were determined to be 6.33×10^3^ RFU and 17.93±1.34 nM, respectively. The flattening of the FRET emission signal, when the acceptor concentration exceeds ∼500 nM, can be attributed to the fact that the increased acceptor concentration gradually saturates the number of donor binding pairs.

The quantitative values of the direct emission signal of Alexa Fluor 488-LFA-1 (F_D_), Alexa Fluor 555-D1-D2-Fc (F_A_) as well as total emission signal (F_DA_) at 570 nm, when the FRET mixtures were excited at 470 nm, along with *a* = 0.129 and *b* = 0.101 were used in Eq. (4) (see “Materials and Methods”) to obtain the FRET emission signal (F_FRET_) at 570 nm for each concentrations of the Alexa Fluor 555-D1-D2-Fc in our FRET binding assay [Fig. 3(a)]. To obtain the maximum FRET emission signal (F_FRETmax_) and the dissociation constant (K_d_), Eq. (6) (see “Materials and Methods”), derived from the nonlinear regression analysis, was used to fit the data sets F_FRET_ vs. Alexa Fluor 555- D1-D2-Fc concentrations as shown in Fig. 5 (adj. R-square = 0.9895). From the analysis, the value of F_FRETmax_ was found to be 6.33×10^3^ RFU and that of K_d_ was 17.93±1.34 nM. This value of K_d_ is in good agreement with that obtained by surface plasmon resonance spectroscopy (SPR) reported by Wu et al. [55]. This method can further be utilized to study the effects of various physiological conditions such as pH, presence of divalent cations on the interaction of LFA-1 and ICAM-1.

### Developing the steady-state FRET based screening assay

#### Determining the optimal acceptor to donor fluorophore (A/D) ratio

The acceptor to donor fluorophore (A/D) ratios were determined using the F/P ratios and the amount of proteins present in the mixture of Alexa Fluor 488-LFA-1 and Alexa Fluor 555-D1-D2-Fc. The optimal A/D ratio is very critical for resonance energy transfer between the acceptor and the donor for a FRET pair. Very high or very low amount of either fluorophores may result in self-quenching and/or insignificant radiative energy transfer. Due to these facts, the A/D ratio that showed the highest energy transfer for our FRET pair of Alexa Fluor 488 and Alexa Fluor 555 was used to develop the steady-state FRET based screening assay to identify the inhibitors of LFA-1 and D1-D2-Fc interaction.

**Figure 6:**
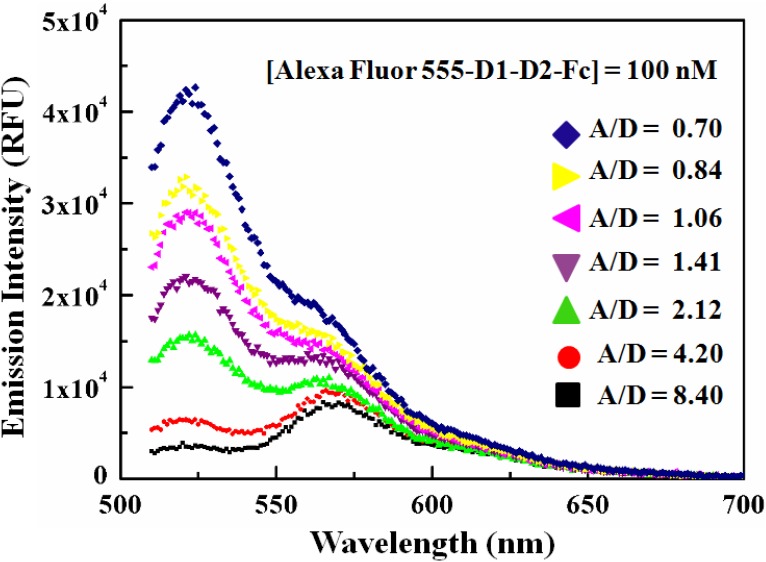
Determination of A/D ratio for optimal FRET between Alexa Fluor 488-LFA-1 and Alexa Fluor 555-D1-D2-Fc. Fluorescence emission scans were obtained for the Alexa Fluor 488-LFA-1 and Alexa Fluor 555-D1-D2-Fc mixture, where the concentration of Alexa Fluor 555-D1-D2-Fc was kept constant at 100 nM and that of Alexa Fluor 488-LFA-1 was varied: 25 nM (A/D = 8.40), 50 nM (A/D = 4.20), 100 nM (A/D = 2.12), 150 nM (A/D = 1.41), 200 nM (A/D = 1.06), 250 nM (A/D = 0.84) and 300 nM (A/D = 0.70). The mixtures were excited at 470 nm. The highest FRET efficiency (∼53.51%) was obtained for A/D = 2.12. For both 4.20 and 8.40 A/D ratios, the donor peak intensity is very small compared to the acceptor peak, while for 2.12 A/D ratio, the donor peak intensity is higher but not overwhelming the acceptor peak intensity. These higher emission counts at 4.20 and 8.40 A/D can be attributed to the direct emission of the acceptor as the acceptor concentration exceeds the saturating concentration required to saturate the donor binding pairs.

To obtain the optimal A/D ratio for our FRET pair, the concentration of the acceptor conjugate, Alexa Fluor 555-D1-D2-Fc was kept constant at 100 nM and that of Alexa Fluor 488-LFA-1 was varied: 25 nM (A/D = 8.40), 50 nM (A/D = 4.20), 100 nM (A/D = 2.12), 150 nM (A/D = 1.41), 200 nM (A/D = 1.06), 250 nM (A/D = 0.84) and 300 nM (A/D = 0.70). The mixtures were excited at 470 nm. The fluorescence emission spectra for all the A/D ratios are shown in Fig. 6. For each A/D ratio, the FRET efficiency was also calculated using Eq. (3) (see “Materials and Methods”) to compare the FRET activity (Table 1). From this we observed that the highest FRET efficiency was achieved at A/D = 2.12) which was used for the subsequent development of our FRET screening assay. This result can be understood from the theoretical investigation by Bojarski et al. [52], which suggested that the FRET efficiency can be significantly increased at a given distance if the energy transfer takes place towards multiple acceptors instead of a single acceptor molecule. Moreover, too little or too much of either fluorophore can also significantly reduce the FRET signals due to self-quenching and/or insufficient energy transfer [56].

**Table 1:** A/D Ratio and FRET Efficiency (%).

A/D Ratio	FRET Efficiency (%): mean ± rms
0.70	4.90±0.79
0.84	0.050±0.003
1.06	16.19±1.29
1.41	0.50±0.01
2.12	53.51±0.10
4.20	4.40±1.01
8.40	0.60±0.03

#### FRET screening assay

Inhibition of the receptor-ligand interaction of LFA-1 and ICAM-1 by short peptide based molecules have been demonstrated for the development of peptide based drugs for diseases related to these interacting protein pairs. In our study, we identified three LFA-1 derived peptides, CD11a_237–261_, CD11a_44–465_, and CD11a_456–465_, to compare their inhibition efficiency via our steady-state FRET based competitive screening assay. Moreover, the inhibition efficiency of lovastatin, a well-known potent inhibitor of the ICAM-1 interaction, was also obtained to further validate the FRET screening platform. Lovastatin belongs to naturally occurring drugs of Statins [34] which has been widely used clinically to lower the serum cholesterol levels [57]. Besides, it has also been shown that lovastatin binds with the highly conserved I-domain of LFA-1 (CD11a I-domain) to inhibit the interaction of LFA-1 with its counter ligand ICAM-1 [58], which leads to a reduction of human immunodeficiency virus type 1 replication [59].

**Figure 7:**
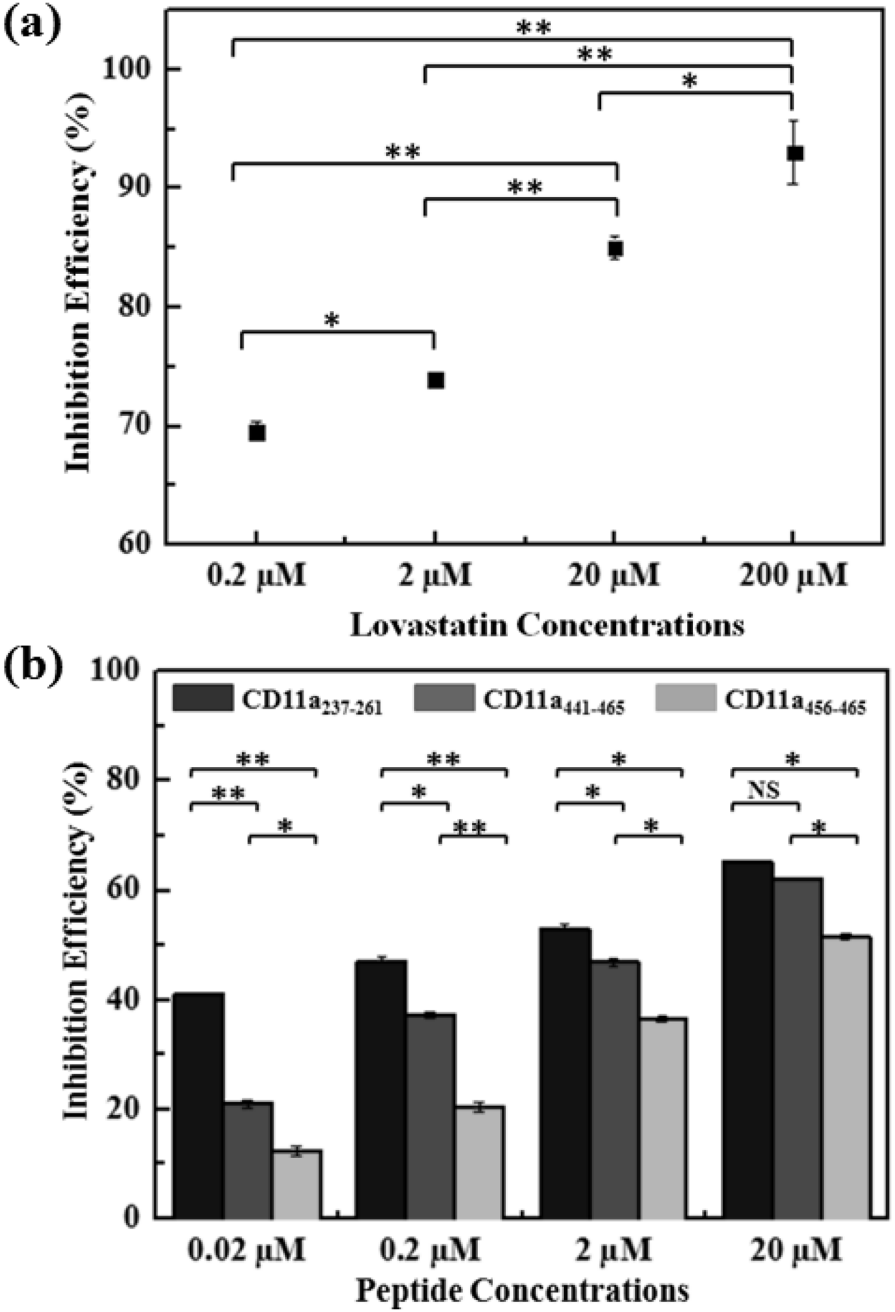
A summary of the inhibitory effects (to the LFA-1/ICAM-1 interactions) of (a) lovastatin; and (b) LFA-1 derived peptides. “*” and “**” denote statistical significance with p<0.05 and p<0.005, respectively, from student t-test. NS: Not Significant. In (a), different concentrations of lovastatin were added to the FRET mixture of Alexa Fluor 488- LFA-1 (100 nM) and Alexa Fluor 555-D1-D2-Fc (100 nM). The inhibition efficiency of lovastatin increased from 69.48±0.77% at 0.2 µM to 90.03±0.06% at 200 µM. This result confirms that this FRET based screening assay is capable of identifying/classifying inhibitors of the LFA-1/ICAM-1 interaction based on inhibition efficiency study. From (b), a comparison of the inhibition efficiencies (to the LFA-1/ICAM-1 interactions) of three LFA-1 derived peptides CD11a_237–261_, CD11a_441–465_, and CD11a_456–465_ indicates that CD11a_237–261_ exhibits the highest inhibition efficiency while CD11a_456–465_ the lowest for all the concentrations tested in this study.



The inhibitory effect of lovastatin is summarized in Fig. 7(a). The FRET efficiency of the FRET mixture, Alexa Fluor 488-LFA-1 (100 nM) and Alexa Fluor 555-D1-D2-Fc (100 nM), was steadily decreased upon incubations with increasing concentrations of the lovastatin (Table S2). In the absence of inhibitors, the FRET efficiency for the equimolar mixture of Alexa Fluor 488-LFA-1 and Alexa Fluor 555-D1-D2-Fc was found to be 53.51%. But with increasing concentration of the lovastatin, the FRET efficiency decreased to 5.3% for 200 µM of lovastatin. The inhibition efficiency (%) was calculated for each concentration of lovastatin using the relation,

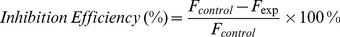
(1)where F_control_ is the FRET efficiency of the Alexa Fluor 488-LFA-1 and Alexa Fluor 555-D1-D2-Fc alone and F_x_ is that of the FRET mixture when incubated with inhibitors. The inhibition efficiency of lovastatin was calculated to be 69.48±0.77%, 73.84±0.56%, 84.98±0.10%, and 90.03±0.06% for 0.2, 2, 20, and 200 µM respectively [Fig. 7(a)]. These results show that the FRET screening assay is sensitive enough to quantify the concentration dependent inhibition activity of inhibitors for the LFA-1 and D1-D2-Fc interaction and can be further used to assess the inhibitors in terms of their inhibition efficiency for the protein interactions of interest.

To characterize and compare the inhibitory activity of the selected peptides, CD11a_237–261_, CD11a_441–465_, and CD11a_456–465_, the FRET mixture was incubated with several concentrations (0.02, 0.2, 2, and 20 µM) of the peptides (following the procedure described in the “Materials and Methods” section) and the FRET efficiency (Tables S3, S4, S5) and the inhibition efficiency [Fig. 7(b)] were calculated for each case. The inhibition efficiency for CD11a_237–261_ at 0.02, 0.2, 2, and 20 µM are 40.81±0.31%, 47.05±0.56%, 53.16±0.70%, 65.13±0.81%, respectively; the corresponding values for CD11a_441–465_, at the same concentrations, are 20.89±0.72%, 37.08±0.29%, 46.93±0.89%, 62.01±0.15%, respectively; and for CD11a_456–465_, the corresponding values are 12.17±0.91%, 20.26±0.69%, 36.46±0.42%, 51.41±0.33%. From these values, we note that the difference in inhibition efficiency of these three peptides is much more significant at low peptide concentration (on the order of 0.02 to 0.2 µM), the cause of which requires further investigation. Nevertheless, in all cases, the inhibition efficiency of CD11a_237–261_ is highest and CD11a_456–465_ the lowest, which are in good agreement with those reported in the literature [36].

## Conclusions

A steady-state ‘in solution’ FRET binding assay has been developed to obtain the dissociation constant (K_d_) of the LFA-1/ICAM-1 interaction using Alexa Fluor 488-LFA-1 as the donor and Alexa Fluor 555-D1-D2-Fc as the acceptor. The dissociation constant for the interaction was determined to be 17.93±1.34 nM. Although the LFA-1/ICAM-1 interaction has been studied extensively, to our knowledge, the application of ‘in solution’ FRET to obtain the dissociation constant of this interaction has not yet been reported. Our approach provides a simple and efficient way to study not only the LFA-1/ICAM-1 interaction kinetics at different biochemical conditions relevant to physiological environment, but also a wide variety of protein-protein interactions in general.

Furthermore, we have also demonstrated a steady-state ‘in-solution’ FRET based screening assay using the same donor and acceptor pair to identify and assess short peptides in terms of their inhibition efficiency to the LFA-1/ICAM-1 interaction. Specifically, we have shown that among the three LFA-1 derived peptides, CD11a_237–261_, CD11a_441–465_, and CD11a_456–465_, CD11a_237–261_ yields the highest inhibition efficiency, and CD11a_456–465_ the lowest. This screening assay can be further developed into a high throughput screening assay to obtain hits from large peptide libraries for the development of potential peptide drugs based on the inhibition of the LFA-1/ICAM-1 interaction.

## Materials and Methods

### Materials

All the reagents were of reagent grade unless otherwise specified. The protein, recombinant human integrin LFA-1 (α_L_β_2_), was purchased from R&D systems (Minneapolis, USA). The human recombinant D1-D2-Fc was synthesized in our lab (Instituto de Carboquímica ICB-CSIC, Zaragoza) in Spain (44). LFA-1 derived peptides, CD11a_237–261_, CD11a_441–465_, and CD11a_456–465_, were purchased from GenScript (Piscataway, USA). The sequences of CD11a_237–261_, CD11a_441–465_, and CD11a_456–465_ were ITDGEATDSGNIDAAKDIIR-YIIGI (LFA-1α derived), GVDVDQDGETEL-IGAPLFYGEQRG (LFA-1α derived), and APLFY- GEQRG (LFA-1α derived), respectively. Mevinolin (*synonym* of Lovastatin) from *Aspergillus species* was obtained from Sigma (Missouri, USA). The fluorophores, Alexa Fluor 488 carboxylic acid, succinimidyl ester and Alexa Fluor 555 carboxylic acid, succinimidyl ester, were procured from Molecular Probes (Eugene, Oregon, USA). The organic solvent, dimethyl sulfoxide (DMSO) dried, was obtained from Merck (Darmstadt, Germany). The Slide-A-Lyzer dialysis cassette G2 (0.5 ml), used for removing free dyes in labelling reactions and exchange of buffer, was bought from Thermo Scientific (Rockford, USA). The 96-microwell transparent/black flat plates were obtained from BD Biosciences (Bedford, MA, USA).

### Methods

In the following sections, the conjugation protocol for the dyes with proteins as well as the scheme of our FRET assay are described in detail.

### Preparation of Alexa Fluor 488- LFA-1 conjugate

The conjugation of Alexa Fluor 488 with LFA-1 was done by following the protocols reported previously [60]. In short, LFA-1 was dissolved in freshly prepared phosphate buffered saline (PBS) at a concentration of 1 mg/ml; 1 mg/ml of Alexa Fluor solution was prepared in DMSO and used immediately. 10 µl of freshly prepared 1 M sodium bicarbonate, pH 9.0, was added to 100 µl of LFA-1 solution and mixed thoroughly. To this solution, 10 µl of Alexa Fluor 488 solution was added and incubated for 2 hrs. in the dark at room temperature under gentle shaking. After the reaction, the free dyes were removed by exhaustive dialysis using Slide-A-lyzer dialysis cassette G2 against PBS buffer, pH 7.4, and left overnight at 4°C.

### Preparation of Alexa Fluor 555-D1-D2-Fc conjugate

The protocol for labelling of Alexa Fluor 555 to D1-D2-Fc was identical to the preparation of Alexa Fluor 488-LFA-1 conjugate, except that the dye was replaced by Alexa Fluor 555 and the starting concentration of D1-D2-Fc solution was 535 µg/ml in PBS.

### Determination of fluorophore to protein (F/P) molar ratio and optimal acceptor to donor fluorophore (A/D) ratio

The fluorophore to protein (F/P) molar ratio, i.e., the ratio of moles of fluorophores to moles of protein in the conjugate, was obtained for each of the two conjugated fluorophore-protein pairs from the known extinction coefficients of the fluorophore and the following equation
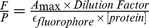
(2)where A_max_ is the absorbance of the fluorophore-protein conjugate at the maximum absorbance wavelength (λ_max_) of the dye, ε_fluorophore_ is the molar extinction coefficient of the fluorophore at λ_max_, and [protein] is the molar concentration of the protein. All the absorption measurements were done using DU 800 spectrophotometer (Beckman coulter, Fullerton, Germany).

The FRET efficiency can be adjusted by changing the fluorophore to protein ratio; the optimal acceptor to donor fluorophore (A/D) ratio was obtained following the procedure reported in the literature [56] with slight modification. In short, A/D is defined as the number of the acceptor fluorophore molecules to donor molecule in the interacting protein complex. Several concentration ratios of Alexa Fluor 488-LFA-1 and Alexa Fluor 555-D1-D2-Fc were incubated for 30 minutes at room temperature and the FRET emission spectra were obtained via a fluorescence multiplate reader, Tecan Infinite M200 pro (Männedorf, Switzerland). For each concentration ratio, the concentration of the acceptor conjugate, Alexa Fluor 555-D1-D2-Fc, was kept constant. The optimal A/D ratio for the FRET pair was selected by comparing the FRET efficiencies for different A/D ratios. FRET activity has been discussed and quantified in different ways in the literatures, including Bossis et al. [61], Stankovic et al. [62], and Lackowicz [40]; however, these methods did not take into account the correction factors for various cross-talks involved in FRET efficiency calculations, and are only suitable for the ideal one donor and one acceptor systems [63]. Due to these facts, we have used equation (3) [64], [65] given below for the FRET efficiency (E) calculation which incorporates the cross-talk corrections for each of the A/D ratios.
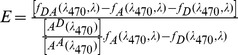
(3)here, (λ_470_, λ) denotes the numerical integral of the emission spectrum between 560 and 610 nm when excited with 470 nm. f_DA_, f_A_, and f_D_ represent the emission spectra of the FRET mixture, acceptor alone, and donor alone, respectively. The absorbance values of donor and acceptor, at the excitation maximum of Alexa Fluor 488 of 470 nm, is represented by A^D^ and A^A^, respectively. Following the definition of overlap integral [40], [51], it follows that increasing the acceptor concentration will lead to an increase in the overall effective acceptor extinction coefficient value; hence, if the spectra are normalized for various concentrations, the overlap integral will remain the same. In this article, the calculation was implemented assuming that there is a change in the overlap integral with different F/P and the (associated) different A/D ratios. The optimal A/D ratio was used for the development of the FRET based screening platform for the assessment of peptides and small molecules for their inhibition efficiency of the LFA-1 and ICAM-1 interactions.

### FRET measurement and dissociation constant (K_d_) determination

To develop the steady-state FRET assay to obtain the dissociation constant (K_d_), the Alexa Fluor 488-LFA-1 was first diluted in PBS, pH 7.4, 1 mM EGTA and 2 mM Mg^2+^ for 30 minutes. In the FRET mixture of total volume 50 µl, the final concentration of Alexa Fluor 488-LFA-1 was 100 nM and that of Alexa Fluor 555-D1-D2-Fc was varied from 0 upto 1.6 µM. The mixtures were transferred into a 96-well plate for high throughput fluorescence emission measurements. The fluorescence emission spectrum of each well was determined with a fluorescence multi-well plate reader Infinite M200 pro (Tecan, Grödig, Austria). The emissions of the FRET mixture at 520 and 570 nm were measured at the donor excitation wavelength of 470 nm, and the emission at 570 nm was measured at the acceptor excitation wavelength of 540 nm. The raw fluorescence emission signals were background-corrected and averaged over three experiments at each specific condition. Each datum point represents the mean (and the associated rms) values of 9 repeated measurements with 3 samples (3 measurements per sample).

In our FRET measurement, two excitation wavelengths of 470 and 530 nm were used to excite Alexa Fluor 488 and Alexa Fluor 555, respectively, in the FRET mixture. When the mixture was excited with 470 nm, two distinct peaks, at 520 and 570 nm, were observed in the FRET emission spectrum. The peak at 520 nm is due to the unquenched Alexa Fluor 488 (F_D_). However, the emission peak intensity at 570 nm (F_DA_) has several components. The analysis of this typical FRET spectrum was done following the procedure reported by Song et al. [49]. In brief, the emission intensity at 570 nm consisted of the contributions from (i) the emission of unquenched Alexa Fluor 488, (ii) the direct emission of Alexa Fluor 555, and (iii) the emission of Alexa Fluor 555 due to the non-radiative energy transfer from Alexa Fluor 488 (F_FRET_). As expected, when the FRET mixture was excited at 540 nm, only a distinct emission peak at 570 nm (F_A_) due to the direct emission of Alexa Fluor 555 was observed. The resultant FRET emission (F_FRET_) was calculated from the following relation,
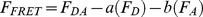
(4)where “*a”* and “*b”* were defined as ratio factors. Here, the ratio “*a*” was defined as the ratio of the emission intensity of Alexa Fluor 488 alone at 570 nm to that at 520 nm when excited at 470 nm, while the ratio “*b*” as the ratio of the emission intensity of Alexa Fluor 555 alone at 570 nm when excited at 470 nm to the intensity at 570 nm when excited at 540 nm. F_FRET_ was calculated for different acceptor to donor ratios and non-linear regression was applied to fit the datasets of F_FRET_ and several concentrations of Alexa Fluor 555-D1-D2-Fc to obtain the maximum FRET emissions (F_FRETmax_). This F_FRETmax_ was eventually used to obtain the dissociation constant (K_d_) of the interaction between D1-D2-Fc and LFA-1 via equation (5) given below, derived from the general law of mass action of protein-protein interactions.
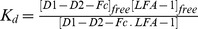
(5)Song *etal.* [49] have reported a systematical methodology to deduce K_d_ from steady-state FRET measurements. Following their procedure and assuming one-to-one interaction between D1-D2-Fc and LFA-1, K_d_ was obtained by fitting the datasets with equation (6),
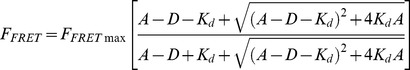
(6)where “A” denotes the concentration of the acceptor, Alexa Fluor 555-D1-D2-Fc, which was varied from 0 to 1.6µM; “D” is the total concentration of donor, Alexa Fluor 488-LFA-1, which was kept fixed at 100 nM.

### FRET screening assay development

In developing steady-state ‘in solution’ FRET based competition assay to assess peptides and small molecules for their inhibition efficiency of the interaction between LFA-1 and D1-D2-Fc, A/D ratio that showed highest energy transfer was used. The donor, Alexa Fluor 488-LFA-1, with a concentration corresponding to the highest A/D ratio was mixed with each kind of LFA-1 derived peptides (CD11a_237–261_, CD11a_441–465_, and CD11a_456–465_) in PBS, pH 7.4, 1 mM EGTA and 2 mM Mg^2+^. These mixtures of Alexa Fluor 488-LFA-1 and peptides were further incubated with Alexa Fluor 555-D1-D2-Fc, the concentration of which was maintained to that of the acceptable A/D ratio, for 30 mins. in dark environment. The total reaction volume for each specific condition was maintained at 50 µl and measurements were done in 96-well plate platform. The FRET efficiency (%) was calculated for each condition for each peptide to compare their inhibition efficiency. For negative control, the FRET efficiency (%) of the mixture of Alexa Fluor 488-LFA-1 and Alexa Fluor 555-D1-D2-Fc, in the absence of the inhibitors, was also obtained. Moreover, as positive control, the FRET efficiency (%) of the aforementioned mixture was obtained in the presence of lovastatin, a potent inhibitor of D1-D2-Fc.

## Supporting Information

Table S1 The key parameters of Tecan Infinite M200 Pro (Männedorf, Switzerland) fluorescence multiple reader for all the measurements, D alone, A alone, and D+A, associated with the experimental results shown in Fig. 2.
(DOCX)

Table S2 FRET efficiency values for the FRET screening assay with lovastatin as the inhibitor for the LFA-1 and D1-D2-Fc interactions.
(DOCX)

Table S3 FRET efficiency values for the FRET screening assay with the peptide CD11a_237–261_ as the inhibitor for the LFA-1 and D1-D2-Fc interactions.
(DOCX)

Table S4 FRET efficiency values for the FRET screening assay with the peptide CD11a_441–465_ as the inhibitor for the LFA-1 and D1-D2-Fc interactions.
(DOCX)

Table S5 FRET efficiency values for the FRET screening assay with the peptide CD11a_456–465_ as the inhibitor for the LFA-1 and D1-D2-Fc interactions.
(DOCX)
