# Supplementary material for: FRET Based Quantification and Screening Technology Platform for the Interactions of Leukocyte Function-Associated Antigen-1 (LFA-1) with InterCellular Adhesion Molecule-1 (ICAM-1)
Source: PLoS One. 2014 Jul 17;9(7):e102572. doi: 10.1371/journal.pone.0102572 (PMC4102529; doi:10.1371/journal.pone.0102572)
Supplement: Table S1 — The key parameters of Tecan Infinite M200 Pro (Männedorf, Switzerland) fluorescence multiple reader for all the measurements, D alone, A alone, and D+A, associated with the experimental results shown in Fig. 2. (DOCX) [file pone.0102572.s001.docx]

**Table S1:** The key parameters of Tecan Infinite M200 Pro (Männedorf, Switzerland) fluorescence multiple reader for all the measurements, D alone, A alone, and D+A, associated with the experimental results shown in Fig. 2

| **Measurement Parameter** | **Instrument Settings** |
| --- | --- |
| Mode | Fluorescence Top |
| Plate | BD Falcon 96 Flat Transparent/Black |
| Excitation wavelength | 470 nm |
| Emission wavelengths scan | 510-700 nm |
| Gain | 100 (manual) |
| Number of flashes | 20 |
| Integration time | 20 µs |
| Lag time | 0 |
| Settle time | 0 |
| Excitation bandwidth | For 316-850 nm: 9 nm (Instrument default) |
| Emission bandwidth | For 280-850 nm: 20 nm (Instrument default) |
