# Supplementary material for: FRET Based Quantification and Screening Technology Platform for the Interactions of Leukocyte Function-Associated Antigen-1 (LFA-1) with InterCellular Adhesion Molecule-1 (ICAM-1)
Source: PLoS One. 2014 Jul 17;9(7):e102572. doi: 10.1371/journal.pone.0102572 (PMC4102529; doi:10.1371/journal.pone.0102572)
Supplement: Table S4 — FRET efficiency values for the FRET screening assay with the peptide CD11a441–465 as the inhibitor for the LFA-1 and D1-D2-Fc interactions. (DOCX) [file pone.0102572.s004.docx]

**Table S4:** FRET efficiency values for the FRET screening assay with the peptide CD11a_441-465_ as the inhibitor for the LFA-1 and D1-D2-Fc interactions

| **CD11a_441-465_ Concentrations (µM)** | **FRET Efficiency (%): mean ± rms** |
| --- | --- |
| 0 | 53.51 ± 0.10 |
| 0.02 | 42.33 ± 0.23 |
| 0.2 | 33.67 ± 0.51 |
| 2 | 28.40 ± 0.02 |
| 20 | 20.33 ± 0.53 |
